# Biomimetic Hybrid Nanocontainers with Selective Permeability

**DOI:** 10.1002/anie.201604677

**Published:** 2016-08-25

**Authors:** Lea Messager, Jonathan R. Burns, Jungyeon Kim, Denis Cecchin, James Hindley, Alice L. B. Pyne, Jens Gaitzsch, Giuseppe Battaglia, Stefan Howorka

**Affiliations:** ^1^ Department of ChemistryInstitute of Structural and Molecular BiologyUniversity College London20 Gordon StreetLondonWC1H OAJUK; ^2^ London Centre of Nanotechnology17–19 Gordon StLondonWC1H 0AHUK

**Keywords:** DNA, enzymes, membranes, nanopores, nanotechnology, polymersomes

## Abstract

Chemistry plays a crucial role in creating synthetic analogues of biomacromolecular structures. Of particular scientific and technological interest are biomimetic vesicles that are inspired by natural membrane compartments and organelles but avoid their drawbacks, such as membrane instability and limited control over cargo transport across the boundaries. In this study, completely synthetic vesicles were developed from stable polymeric walls and easy‐to‐engineer membrane DNA nanopores. The hybrid nanocontainers feature selective permeability and permit the transport of organic molecules of 1.5 nm size. Larger enzymes (ca. 5 nm) can be encapsulated and retained within the vesicles yet remain catalytically active. The hybrid structures constitute a new type of enzymatic nanoreactor. The high tunability of the polymeric vesicles and DNA pores will be key in tailoring the nanocontainers for applications in drug delivery, bioimaging, biocatalysis, and cell mimicry.

The quest to build chemically controlled bioinspired structures focuses increasingly on multicomponent systems. One ambitious target is to create membrane‐enclosed vesicles that control the exchange of cargo between the interior and the environment, yet encapsulate other active materials such as enzymes and fluorescent proteins.^1^ These rationally designed structures could find applications in synthetic biology, biotechnology, and biomedicine.

One of the most powerful approaches to build synthetic vesicles involves polymers. Polymersomes have membranes composed of amphiphilic block copolymers with tunable mechanical properties and thickness.^2^ The synthetic nature of the building blocks allows to engineer permeable membranes to enable exchange of matter with the environment.^2^, ^3^ Alternatively, the polymersome membranes can be punctured with peptide or protein channels to help achieve more selective exchange of ions^4^ or small organic molecules, for example.3h, 5 However, protein pores are defined by their biological origin with consequent limitation on the design of cargo transport. Furthermore, most membrane proteins are structurally fragile. Hence, very few natural pores possess the required robustness to survive reconstitution within synthetic vesicles.

Recently developed synthetic membrane‐spanning DNA nanopores provide a new and potentially generic route for controlled transport across membranes.^6^ Like all rationally designed DNA nanostructures, DNA pores can be easily fabricated through the self‐assembly of component oligonucleotides. The modular construction principle has enabled customized pore diameters^7^ and installation of a controllable gate to regulate transport.6d The negatively charged DNA pores carry hydrophobic membrane anchors for membrane insertion. DNA pores have so far only been placed into lipid bilayer membranes,6a–6f and it is not known whether they also anchor into polymersomes to form membrane‐puncturing nanosized holes.

In this work, we created organelle‐inspired synthetic hybrid nanocontainers composed of polymersomes and DNA nanopores (Figure 1). The nanocontainers exhibit designed size‐dependent permeability and facilitate the transport of enzyme substrates across the nanoporous membrane while the larger enzymes are retained (Figure 1).

**Figure 1:**
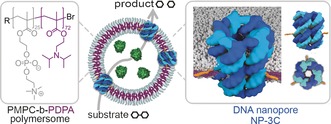
Functional hybrid nanocontainers composed of polymersomes (middle) are assembled from amphiphilic block copolymers (left) and membrane‐spanning DNA nanopores (right). The containers display size‐selective permeability; they permit the transport of organic enzyme substrates and products through the DNA nanopores but retain bioactive encapsulated enzymes.

The polymersomes were formed from the amphiphilic block copolymer poly 2‐(methacryloyloxy)ethyl phosphorylcholine‐b‐disisopropylamino) ethyl methacrylate (PMPC‐b‐PDPA; Figure 1). The polymer was synthesized through atom‐transfer radical polymerization (ATRP; Scheme S1A in the Supporting Information) at a stoichiometry of PMPC_25_‐b‐PDPA_72_ and with a homogenous size distribution (polydispersity index (PDI) of 1.12), as determined by ^1^H NMR (Figure S2) and size‐exclusion chromatography (SEC, not shown). Polymersomes were obtained through self‐assembly of PMPC_25_‐b‐PDPA_72_ by thin‐film hydration and subsequent purification by centrifugation. The polymersomes were of homogeneous spherical shape with a hydrodynamic diameter between 100 and 200 nm (PDI=0.15), as established by transmission electron microscopy (TEM) and dynamic light scattering (DLS; Figures 2 A and Figure S3). Scanning transmission electron microscopy confirmed that the polymersome membrane had a thickness of 6.5±1.2 nm (*n*=10; Figure S4), in accordance with previously published results.^8^

**Figure 2:**
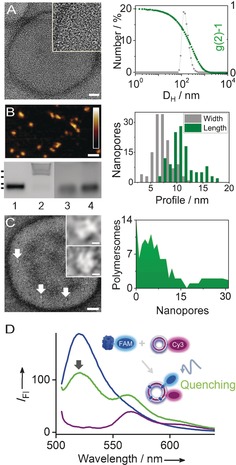
Characterization of polymersomes, DNA nanopores, and hybrid nanocontainers with membrane‐spanning pores. A) Analysis of AM‐stained PMPC_25_‐PDPA_72_ polymersomes by TEM (left panel) and DLS (right panel). The scale bar for the TEM image is 20 nm, and the inset shows a 2‐fold magnification. B) Agarose gel electrophoresis (left, bottom panel) confirms the assembly of the DNA nanopores NP‐0C and NP‐3C (lanes 1, 3 and 2, 4, respectively) without (lanes 1, 2) or with (lanes 3, 4) 0.23 % SDS (v/v). The dots at the left of the gel indicate the position of the dsDNA markers for 10, 3, 1, and 0.5 kbp. An AFM micrograph of NP‐0C adsorbed on mica (left, top panel; scale bar, 20 nm; vertical scale 1.8 nm, scale bar inset) reveals the pore dimensions as summarized in a histogram (right panel) for width and length, where length is equivalent to the height of an upright DNA pore. C) Detection of NP‐3C in a polymersome as bright spots in TEM images after AM staining (left panel; scale bar, 25 nm; inset 2 nm) and analysis of the bright spots from 110 polymersomes (right panel). D) Fluorescence spectra of FAM‐labeled NP‐3C at 0.5 μm (blue), and Cy3‐PMPC_25_‐PDPA_72_ polymersomes at 2.5 mg mL^−1^ (violet) and a mixture of both (green) at the same concentration acquired at *λ*_exc_=495 nm. *I*_Fl_=fluorescence emission intensity.

To build designed holes into polymersome walls, the DNA nanopore NP‐3C, which has outer dimensions of 9 nm×6 nm and a lumen diameter of 2 nm, was used (Figure 1). NP‐3C is composed of six interconnected DNA duplexes and carries at its outside perimeter three cholesterol tags for membrane insertion (Figure 1; 2D DNA map, sequences of six oligonucleotides; Figure S1 and Table S1).6d A second pore without cholesterol anchors, NP‐0C, served as a negative control. The two DNA nanopores were successfully assembled as shown by agarose gel electrophoresis (Figure 2 B, left bottom panel; Figure S5). NP‐3C migrated higher than NP‐0C (Figure 2 B, lanes 2 and 1, respectively) due to hydrophobic interaction with the gel matrix which could, however, be reduced by adding detergent SDS (Figure 2 B, lanes 4 and 3).6d

The size of the nanopores was determined by atomic force microscopy (AFM; Figure 2 B, left top panel). The elongated features in AFM micrographs represent DNA pores that are oriented with their vertical axis parallel on the substrate. Their pores had an average length and width of 11.3±3.2 nm and 7.4±1.7 nm, respectively (n=131; Figure 2 B, right panel; Figure S6), which is within the nominal pore dimensions of 9×5 nm.6d Additional analysis by TEM after staining with uranyl acetate (UA; Figure S7, S8) and ammonium molybdate (AM; Figure S9) confirmed the AFM results on the expected size of the DNA pores.

Hybrid nanocontainers were formed by inserting the DNA nanopore NP‐3C into the walls of polymersomes (Figure 1) through incubation. The mechanism for insertion has not yet been confirmed but likely involves a first step of membrane tethering, followed in a second step by complete insertion. The resulting polymersomes showed bright spots in the TEM analysis (Figure 2 C, left panel; AM stain), which represent wall‐anchored pores. No similar features were found for polymersome‐only samples (Figure 2 A) or polymersomes incubated with the anchor‐free NP‐0C (Figure S10). The TEM image was subjected to fast Fourier transform (FFT) filtering to highlight the pores within the polymersome membrane (Figure S11). Representative FFT images of pores show a ring of high density (Figure 2 C, right) that probably reflects the six hexagonally arranged DNA duplexes. Analysis of 110 vesicles established that 87 % of the polymersomes bear NP‐3C nanopores, with an average of 7 pores per vesicle (Figure 2 C, right panel). Incubating the polymersomes with a higher concentration of nanopores led to more pore insertion (see below). Rupturing and fragmentation of the polymersomes by the nanopores was not observed.

The insertion of pores was also confirmed by fluorescence measurements of aqueous dispersions of vesicles (Figure 2 D). For this analysis, the NP‐3C pore carried the fluorescein dye FAM (blue in Figure 2 D; *λ*_exc_=495 nm, *λ*_emm_=520 nm); fluorophores can be quenched when inserted into hydrophobic membrane environments.^9^ The polymer of the vesicles was conjugated to the Cy3 dye (purple in Figure 2 D; *λ*_exc_=550 nm, *λ*_emm_=570 nm) to enable fluorescence resonance energy transfer (FRET) with FAM and thereby provide additional experimental proof for membrane anchoring. Details of the chemical linkage between fluorophores and the molecular components, and the amount and purity of the labeled polymer are available in Table S1, Schemes S1B, C, and Figure S12. UV/Vis spectra of isolated FAM‐nanopores and Cy3‐polymersomes featured the expected single emission peaks at 520 and 570 nm, respectively (Figure 2 D, blue and purple). Mixing the two molecular components yielded a decreased FAM signal at 520 nm (Figure 2 D, green), which indicates quenching owing to membrane insertion. Quenching is the dominating molecular reason since a very similar drop in the FAM signal was also observed for unlabeled polymersomes (Figure S13). Using Cy3‐labeled polymersomes did not uncover a clear FRET effect since the signal at 570 nm (Figure 2 D, green) was mostly due to the inherent Cy3 fluorescence of the labeled polymersomes (Figure 2 D, purple). Varying the ratio of pores to polymersomes in the incubation mixture led to shifts in the fluorescence signal expected for tunable amounts of anchored pores (Figure S13).

The functionality of the nanopore‐punctured polymersomes was demonstrated with an enzymatic assay that also demonstrates that the hybrid containers can be turned into enzymatic nanoreacters (Figure 3 A). The assay relied on the transport of fluorogenic enzyme substrate B‐NAR‐AMC through the DNA pores and its cleavage to the fluorescent product AMC by polymersome‐encapsulated trypsin (Figure 3 A). The enzyme substrate has a maximum length of 1.5 nm calculated for an energy‐minimized structure^10^ and features a positive charge (Figure 3 B). The substrate was deliberately chosen to probe whether it can pass the 2 nm DNA nanopore. Smaller 1 nm organic molecules with positive charge are known to permeate through the pore.6d

**Figure 3:**
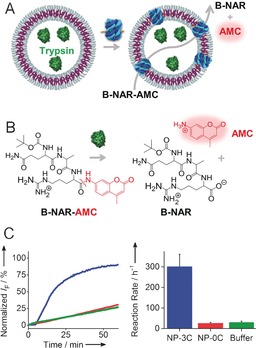
Polymersomes with membrane‐inserted DNA nanopores retain enzymes but are permeable to smaller enzyme substrates and products. A) Schematic representation of a polymersome with encapsulated hydrolytic enzyme trypsin, which cleaves the substrate B‐NAR‐AMC to release the fluorescent product AMC. B) Structure of substrate peptide B‐NAR‐AMC (Boc‐Gln‐Ala‐Arg‐7‐amido‐4‐methylcoumarin) and its hydrolysis by trypsin. C) Kinetic fluorescence traces of the nanocontainer with the NP‐3C DNA nanopores (blue), and negative controls with NP‐0C (red) or without nanopores (green). The approximate molecular ratio of peptide/enzyme/polymersome/DNA pore was 80 000:280:1:>7. The number of DNA pores is based on the TEM analysis in Figure 2 but is most likely higher since an 8‐fold higher molar ratio of DNA pores to polymersomes was used in the incubation mixture for the enzymatic assay compared to the preparation of samples for the TEM analysis.

Enzyme‐filled nanocontainers were obtained by encapsulating trypsin inside the PMPC‐b‐PDPA polymersomes through electroporation.^11^ This procedure did not affect the polymersome diameter or structural integrity (DLS and TEM analysis, Figure S14). Purification of trypsin‐containing vesicles by SEC (Figure S15) and measurement of absorbance at 280 nm and 220 nm established the protein and polymer concentration, respectively (Figures S16–S18, Table S2). The ratio of the concentrations yielded an average of 280 encapsulated enzyme molecules per polymersome (Table S2).

The walls of enzyme‐filled nanocontainers were punctured with DNA nanopore NP‐3C, and the assay for pore transport was initiated by adding fluorogenic B‐NAR‐AMC to the polymersome dispersion (Figure 3 A) and tracking the enzymatic release of AMC by measuring the fluorescence emission at 440 nm. The kinetic trace (Figure 3 C, blue line) reached a maximum within 30 min, thus implying successful transport through the DNA pores.

In support of nanopore‐facilitated transport, 10‐fold slower kinetics were observed for nanocontainers incubated with non‐anchored NP‐0C (Figure 3 C, red) or no pore (Figure 3 C, green). Both negative controls with minimal transport indicate that the membrane is not completely impermeable for the substrate, which contains hydrophobic methylcoumarin and two hydrophobic amino acids. Nonspecific transport is an inherent characteristic of many other amphiphatic fluorogenic substrates.^12^ The trace for the positive control comprising trypsin and substrate but no polymersome showed much faster kinetics (Figures S19 and S20, Table S3). The overall kinetics for signal generation hence comprise 1) the rate‐defining transport of substrate through the DNA nanopores and to a minor extent across the polymersome wall, and 2) the fast and non‐rate‐limiting turnover of the substrate by the encapsulated trypsin. A high catalytic efficiency of 2.9×10^7^ m ^−1^ s^−1^ and a high *k*_cat_ value of 120 s^−1^ have been reported for trypsin with the peptide substrate,^10^ but the values can vary depending on the source of trypsin.

In summary, we have demonstrated the creation of synthetic, biomimetic vesicles composed of polymer walls and artificial membrane‐spanning pores made of DNA. The nanocontainers have designed functionality and exhibit size‐dependent permeability. The transport of peptides through DNA nanopores is enabled, while large enzymes are retained. The hybrid structures are also new. Previously, either DNA pores were inserted in bilayer vesicles, or polymersomes were combined with protein pores. Our results support the future development of more advanced synthetic nanoreactors that combine the chemical flexibility of polymersomes with the rational design of stimulus‐responsive DNA pores to control transport of cargo.

## Supporting information

As a service to our authors and readers, this journal provides supporting information supplied by the authors. Such materials are peer reviewed and may be re‐organized for online delivery, but are not copy‐edited or typeset. Technical support issues arising from supporting information (other than missing files) should be addressed to the authors.

Supplementary
