# Supplementary material for: Biomimetic Hybrid Nanocontainers with Selective Permeability
Source: Angew Chem Int Ed Engl. 2016 Aug 25;55(37):11106–9. doi: 10.1002/anie.201604677 (PMC5103200; doi:10.1002/anie.201604677)
Supplement: Supplementary file 1 — Supplementary [file ANIE-55-11106-s001.pdf]

Angewandte Chemie
Eine Zeitschrift der Gesellschaft Deutscher Chemiker

Supporting Information

Biomimetic Hybrid Nanocontainers with Selective Permeability

Lea Messager[+], Jonathan R. Burns[+], Jungyeon Kim[+], Denis Cecchin, James Hindley, Alice L. B. Pyne, Jens Gaitzsch, Giuseppe Battaglia, and Stefan Howorka**

anie_201604677_sm_miscellaneous_information.pdf

Supporting Information

Biomimetic Hybrid Nanocontainers of Designed Permeability

Lea Messager, Jonathan R. Burns, Jungyeon Kim, Denis Cecchin, James Hindley, Alice Pyne, Jens Gaitzsch, Giuseppe Battaglia, and Stefan Howorka

1. Materials and Methods 2
   1.1. Materials 2
   1.2. Synthesis of $PMPC_{25}$-$PDPA_{72}$ by ATRP 2
   1.3. Synthesis of $PDPA_{70}$-$PMPC_{25}$-S-S-$PMPC_{25}$-$PDPA_{70}$ by ATRP 3
   1.4. Synthesis of Cy3-Labeled $PMPC_{25}$-$PDPA_{70}$ 3
   1.5. Preparation of Polymersomes 4
   1.6. Assembly and Gel Characterization of DNA Nanopores 5
   1.7. AFM Analysis of DNA Nanopores 6
   1.8. Dynamic Light Scattering Analysis of Polymersomes 7
   1.9. Transmission Electron Microscopy of DNA Nanopores and Polymersomes 7
   1.10. Fluorescence Measurements of DNA Nanopores and Polymersomes 8
   1.11. Encapsulation of Trypsin into Polymersomes, their Purification by Size Exclusion Chromatography and Characterization by UV-vis Spectroscopy 8
   1.12. Enzymatic Assays of Polymersomes with Encapsulated Trypsin 9
2. Experimental Results 10
   2.1. Characterization of Polymersomes 10
   2.2. Characterization of DNA Nanopores 14
   2.3. Characterization of Hybrid Nanocontainers Composed of Polymersomes and DNA Nanopores 18
   2.4. Characterization of Nanocontainers with Encapsulated Enzymes 21
   2.5. Characterization of Hybrid Nanocontainers with Enzymatic Assays 24

1. Materials and Methods

1.1. Materials

2-(Methacryloyloxy)ethyl phosphorylcholine monomer (MPC, 99.9% purity) was kindly donated by Biocompatibles U.K. Unmodified, fluorescein amidite (FAM)-labeled, and cholesterol-labeled DNA oligonucleotides were procured from Integrated DNA Technologies (Leuven, Belgium) on a 1 µmol scale with HPLC or PAGE purification. Triphenylphosphine (PPh_3) was purchased from Alfa Aesar (Heysham, UK). Cy3-maleimide (Cy3-mal) was obtained from Lumiprobe (Hannover, Germany). HPLC-grade dichloromethane (CH_2Cl_2), chloroform ($CHCl_3$) and semi-permeable cellulose dialysis tubing (Spectra/Por 6 MWCO 1,000) were purchased from Fisher Scientific (Loughborough, UK). Silica gel 60 (0.063 - 0.200 µm) used for removal of ATRP catalyst or unlabeled Cy3-mal was purchased from Merck (Darmstadt, Germany). The remaining chemicals were bought from Sigma Aldrich and used as received: 2-(diisopropylamino)ethyl methacrylate (DPA), copper(I) bromide (Cu(I)Br, 99.999%), 2,2′-bipyridine (bpy, 99%), bis[2-(2-bromoisobutyryloxy)ethyl] disulfide ($BiBOE_2S_2$), anhydrous ethanol (EtOH, 99%), anhydrous methanol (MeOH, ≥ 99.8%), tetrafluoroacetic acid (TFA), phosphotungstic acid (PTA), ammonium molybdate (AM), PBS tablets, trypsin from porcine pancreas (1000-2000 UI/mg), sepharose 4B, and Boc-Gln-Ala-Arg-7-amido-4-methylcoumarin hydrochloride (B-NAR-AMC).

1.2. Synthesis of $PMPC_{25}$-$PDPA_{72}$ by ATRP

Block copolymer PMPC-PDPA was synthesized by atom-transfer radical-polymerization (ATRP) following a published protocol.[1] Briefly, a solution in morpholinoethyl-bromoisobutyric acid ester (ME-Br, synthesis described previously)[2] (0.190 g, 0.68 mmol, 1 eq.) in EtOH (5 mL) was placed in a round-bottom flask before addition of MPC (5.000 g, 1.70 mmol, 25 eq.). The mixture was stirred and further purged with nitrogen for 30 min and heated to 30 °C. Then, a mixture of bpy (0.223 g, 1.42 mmol, 2 eq.) and Cu(I)Br (0.097 g, 0.68 mmol, 1 eq.) was added under a constant nitrogen flow. The mixture was stirred for 60 min to yield a highly viscous brown substance and sampled with NMR to estimate the extent of conversion. Meanwhile, a solution of DPA (12.27 g, 57.6 mmol, 85 eq.) in EtOH (13 mL) was prepared and purged with nitrogen for 60 min in a separate flask. DPA solution was added to the polymerization mixture, and the reaction solution was purged for another 10 min and left overnight at 30°C. After 18 h, ^1H NMR analysis confirmed that the conversion was >99%. Upon diluting the reaction mixture in EtOH (30 mL), the solution gradually turned green, indicating oxidation of the copper-based catalyst. The mixture was passed through silica and the solvent was partially evaporated to give an opaque solution which was then dialyzed (MWCO 1,000 Da) against CH_2Cl_2, MeOH, and water (2 times each) for 8 - 14 h for

each dialysis cycle. The polymer was freeze-dried and dried at 120 °C for 2 h under vacuum resulting in a yield of 13.3 g (77%). ^{1}H NMR analysis in mixture of $CDCl_3$/MeOD (3:1) determined the composition of the polymer to be $PMPC_{25}$-$PDPA_{72}$. Size-exclusion chromatography (SEC) established that the polydispersity index (PDI) had a value of 1.22.

1.3. Synthesis of $PDPA_{70}$-$PMPC_{25}$-S-S-$PMPC_{25}$-$PDPA_{70}$ by ATRP

A similar synthetic procedure to $PMPC_{25}$-$PDPA_{72}$ was used, but with $BiBOE_2S_2$ as initiator.[3] Briefly, a solution of $BiBOE_2S_2$ (0.1850 g, 0.40 mmol, 1 eq.) in anhydrous EtOH (4 mL) was transferred to a flask containing MPC (6.045 g, 20 mmol, 50 eq.) under nitrogen. The resulting solution was purged with nitrogen for 35 min. Then, a mixture of bpy (0.2543 g, 1.63 mmol, 4 eq.) and Cu(I)Br (0.1169 g, 0.81 mmol, 2 eq.) was added under a constant flow of nitrogen. The mixture was stirred for 60 min to yield a highly viscous brown substance and sampled with ^{1}H NMR to estimate the extent of conversion. A solution of DPA (12.130 g, 56.9 mmol, 142 eq.) in anhydrous EtOH (14 mL) was prepared and purged with nitrogen for 60 min in a separate flask. Then, the DPA solution was added to the polymerization mixture and the reaction mixture was purged for another 10 min and left overnight at 30°C. After 18 h, ^{1}H NMR analysis confirmed that the conversion was > 99% and the reaction mixture was diluted in EtOH. The solution gradually turned green, indicating oxidation of the copper-based catalyst system. The mixture was passed through silica, and the solvent was partially evaporated to give an opaque solution which was dialyzed (MWCO 1,000 Da) against a mixture of CH_2Cl_2, MeOH, and water (2 times each) for 8 - 14 h for each dialysis cycle. The polymer was first freeze-dried and then dried at 120 °C for 2 h under vacuum resulting in a yield of 16.3 g (92%). Analysis via ^{1}H NMR in $CDCl_3$ and MeOD (3:1) determined the composition to be $PDPA_{70}$-$PMPC_{25}$-S-S-$PMPC_{25}$-$PDPA_{70}$. The SEC-derived PDI had a value of 1.24.

1.4. Synthesis of Cy3-Labeled $PMPC_{25}$-$PDPA_{70}$

Block copolymer $PMPC_{25}$-$PDPA_{70}$ was labeled with the Cy3 fluorophore[3] for fluorescence measurements. $PDPA_{70}$-$PMPC_{25}$-S-S-$PMPC_{25}$-$PDPA_{70}$ (350 mg, 7.8 µmole, 1eq.) was dissolved in a mixture of $CHCl_3$ and MeOH (2:1) in the presence of Cy3-maleimide (12.0 mg; 17.1 µmole, 2.2 eq.) within a round-bottom flask. After complete polymer dissolution, PPh_3 (2.6 mg; 10.1 µmole; 1.3 eq.) was added to reduce the disulfide bond of the polymer and initiate the reaction. The mixture was stirred for 48 h and then filtrated through silica and rinsed thoroughly with EtOH to remove excess Cy3-mal. The solution was further purified by dialysis (MWCO 1,000 Da) twice each against EtOH and water (8 to 14 h per cycle) and then freeze-dried. The incorporation of Cy3 into the block copolymer was confirmed by analysis

with HPLC. The HPLC Dionex Ultimate 3000 system from Thermo Scientific was equipped with an autosampler, a pressure pump, an FLD-fluorescence emission detector, and a Jupiter 5 µm C18 column from Phenomenex. The mobile phase was composed of (A) water (0.05% TFA) and (B) MeOH (0.05% TFA) using a gradient of 5 – 80% B over 120 min. The flow rate was 1 mL min^{-1}, the detection was monitored at an absorption wavelength of 568 nm, and the injection volume was 20 µL. The reaction achieved full conversion to Cy3-$PMPC_{25}$-$PDPA_{70}$ and no free dye was detected.

1.5. Preparation of Polymersomes

All polymersome dispersions were prepared by thin film hydration.[4] Typically, block copolymer $PMPC_{25}$-$PDPA_{72}$, or a mixture of $PMPC_{25}$-$PDPA_{72}$ and Cy3-$PMPC_{25}$-$PDPA_{72}$ (95:5, 25 mg) was dissolved in a mixture of $CHCl_3$ and MeOH (2:1, 5 mL) and then dried under vacuum at 30°C overnight. The resulting dried polymer film was hydrated in PBS (5 mL) and stirred vigorously. After 7 weeks under stirring, the polymeric vesicle suspension was purified through centrifugation cycles. Briefly, an aliquot (500 µL) was centrifuged for 10 min at 1000 RCF. The supernatant was further centrifuged for 20 min at 23 000 RCF, and the resulting pellet was re-suspended in PBS (450 µL) and sonicated for 20 min, yielding a monodisperse suspension of polymeric vesicles. The concentration of $PMPC_{25}$-$PDPA_{72}$ within polymersome suspension was measured by UV-vis spectroscopy. Typically, a polymersome suspension (20 µL) was diluted 1:10 in PBS, pH 2.0, and the absorbance was recorded at λ = 220 nm. The concentration of $PMPC_{25}$-$PDPA_{72}$ was calculated using a calibration curve of the polymer. The polymersome size and its distribution were determined by dynamic light scattering as described in section 1.8. To calculate the number of polymersomes in a unit volume of suspension, the concentration of $PMPC_{25}$-$PDPA_{72}$ and the polymersome size were used as input variables for a Matlab® script which is based on several equations. Following equation (1), the number of polymersomes, N_p, is given by the experimentally determined total number of polymer chains, N_c, divided by the number of polymer chains per polymersome, N_{agg}.

$$N_p = \frac{N_c}{N_{agg}} \quad (1)$$

N_{agg} is defined in equation (2) as the ratio between the volume of the polymersome hydrophobic membrane, V_p, divided by the product of molecular volume of the PDPA block of the polymer, V_{PDPA}, assuming a vesicle packing parameter p = 1.

$$N_{agg} = \frac{V_p}{V_{PDPA}} \quad (2)$$

The molecular volume , V_{PDPA}, is defined as:

$$V_{PDPA} = \frac{M_{PDPA}}{\rho_{PDPA} N_A} \quad (3)$$

where M_{PDPA} is the molar mass of the PDPA block ρ_{PDPA} the bulk density of the hydrophobic chain and is 1.02 g/ml and, N_A, is the Avogadro number.

1.6. Assembly and Gel Characterization of DNA Nanopores

DNA nanopores with cholesterol tags (NP-3C) and without cholesterol tags (NP-0C) (2D map in Figure S1) were assembled from an equimolar mixture of six DNA strands with sequences listed in Table S1. For NP-0C, oligonucleotides 1-6 were used, and for NP-3C the oligonucleotides were 1(chol), 2, 3(chol), 4, 5(chol), 6. For the optional fluorescence-tagged DNA pore, oligo 2 was replaced with 2(F) carrying a FAM modification. The equimolar sets of six oligonucleotides (1 µM each, 1 mL total volume) were dissolved in 0.3 M KCl, 15 mM Tris, pH 8.0 or PBS, pH 7.4, as indicated. The mixture was heated to 95°C and cooled to 20°C at a rate of 0.5 °C min^{-1} using a PCR thermocycler. The successful assembly was confirmed by agarose gel electrophoresis.

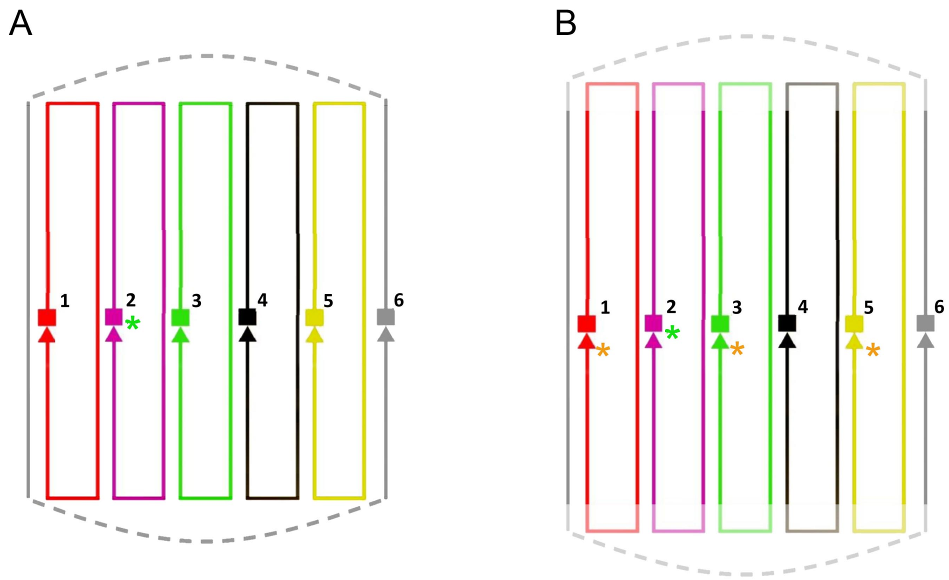

Figure S1. 2D maps of DNA nanopores, (A) NP-0 and (B) NP-3C. The component DNA strands as represented as lines, and the 5' and 3' termini of the strands are indicated by squares and triangles, respectively. Mis-matched T_4 hairpin loops are of semi-transparent color. The positions for the cholesterol modifications are indicated by orange asterisks, and the position of the FAM modification is indicated by a green asterisk.

Table S1. Names, chemical modifications, and sequences of DNA oligonucleotides used to prepare DNA nanopores NP-0C, NP-3C and FAM-labeled NP-3C.

ID	Sequence 5' → 3'
1	AGCGAACGTGGATTTTGTCCGACATCGGCAAGCTCCCTTTTTCGACTATT
2	CCGATGTCGGACTTTTACACGATCTTCGCCTGCTGGGTTTTGGGAGCTTG
2(F)	FAM-CCGATGTCGGACTTTTACACGATCTTCGCCTGCTGGGTTTTGGGAGCTTG
3	CGAAGATCGTGTTTTTCCACAGTTGATTGCCCTTCACTTTTCCCAGCAGG
4	AATCAACTGTGGTTTTTCTCACTGGTGATTAGAATGCTTTTGTGAAGGGC
5	TCACCAGTGAGATTTTTGTCGTACCAGGTGCATGGATTTTTGCATTCTAA
6	CCTGGTACGACATTTTTCCACGTTCGCTAATAGTCGATTTTATCCATGCA
1(chol)	Sequence of 1 carrying a cholesterol via tri(ethylene glycol) TEG linker at the 3' end
3(chol)	Sequence of 3 carrying a cholesterol via a TEG linker at the 3' terminus
5(chol)	Sequence of 5 carrying a cholesterol via a TEG linker at the 3' terminus

1.7. AFM Analysis of DNA Nanopores

DNA nanopores NP-0C were analyzed by AFM following a published approach that involves adsorption using divalent cations ($NiCl_2$)[5-6] to bridge the negative charges of mica and the negatively charged DNA nanostructures. A solution of nanopores (0.005 µM, 25 µL, in 10 mM Tris, 10 mM $NiCl_2$ pH 7.4) was added to freshly cleaved mica and incubated for 5 min. The supernatant was then removed followed by the addition of buffer solution (25 µL, 10 mM Tris, pH 7.4) reducing the $NiCl_2$ concentration to ~1 mM. AFM topographical images were acquired in fluid at RT using a Multimode 8 Atomic Force Microscope (Bruker Santa Barbara, CA, USA) operated in PeakForce Tapping mode using a MSNL-E cantilever (k~0.1 N m^{-1}). Imaging was performed at a setpoint of 0.014 Volts (approximately 60 pN) and at a PeakForce frequency of 2 kHz with a 5 nm PeakForce amplitude. Images were processed using Gwyddion[7] for line-by-line flattening and removal of tilt using a first order polynomial, and the color scale was set to 1.8 nm to show individual nanopores against the background (Figure 2B, Figure S6-A). Line profiles were taken in Gwyddion along the long (length) and short (width) axis of NP-0C as shown by the grey and green lines in Figure S6-A, and plotted in Origin (OriginLab) (Figure S6-B). The dimensions of the nanopore were determined by this method as the full-width-at-half-maximum (FWHM) of a Gaussian peak fitted to the profiles. Singular pores were selected for statistical analysis by elevation using Gwyddion (Figure S6-

C). The grain distribution function was used to determine the width (minimum bounding size) and height (maximum bounding size) of the selected nanopores and plotted in Origin (Figure 2B, Figure S6-D).

1.8. Dynamic Light Scattering Analysis of Polymersomes

The size distributions of polymersomes and DNA nanopores were analyzed by dynamic light scattering (DLS) using a Malvern Zetasizer NanoZS instrument operated with a He-Ne 633 nm laser and a detector set at an angle of 173°. For each measurement, 20 µL of sample was diluted in filtered PBS (1 mL, 0.2 µm filter). The zeta-averaged hydrodynamic diameter, D_H, was calculated from diffusion coefficients using the Stokes–Einstein equation. Correlogram analysis was performed with software from Malvern. The particle size distribution was calculated by the cumulant analysis method.

1.9. Transmission Electron Microscopy of DNA Nanopores and Polymersomes

The morphology of DNA nanopores, $PMPC_{25}$-$PDPA_{72}$ polymersomes, and mixture of both were analyzed by transmission electron microscopy (TEM) using a JEOL 2100 instrument operated at 200 kV and equipped with a CCD camera from Gatan. Polymersomes (0.5 mg mL^{-1}, 5 µL, in PBS, pH 7.4), DNA nanopores (2 – 50 nM, 5 µL, in 0.2 x PBS, pH 7.4), or a mixture of both were deposited for 1 min onto a freshly glow-discharged (SC7620, Quorum Technologies) copper-coated carbon TEM grid (Agar Technologies) and subsequently blotted with filter paper. Next, 20 µL of water, followed by 20 µL of staining solution was deposited on the carbon grid, with a blotting step between each deposition. The samples were stained with phoshotungstic acid (PTA), uranyl acetate (UA), or ammonium molybdate (AM), depending on the nature of the sample. The staining solutions were freshly prepared. For PTA, a solution (0.75 wt%) was prepared by diluting PTA (37.5 mg) into boiling ultrapure water (12.5 mL). The pH was adjusted to 7.0 by adding NaOH (5 M), and the solution was filtered twice before storing it at 4 °C. A solution of UA (1 wt%) was prepared with ultrapure water and filtration. The solution of AM (2 wt%) was obtained with ultrapure water and adjusting to pH 7.0 using a fresh solution of ammonia (5 M). Membrane thickness was measured by switching from TEM imaging to scanning transmission electron microscopy (STEM) imaging,[1] using the JEOL 2100 microscope. Polymersomes stained with PTA were first imaged by conventional TEM, followed by analysis in STEM using the dark-field mode to map the distribution of tungsten within the vesicle. Plot profiles were then taken with Image J across the membrane and the FWHM was taken.

1.10. Fluorescence Measurements of DNA Nanopores and Polymersomes

Fluorescent measurements were carried out on a Varian Carry Eclipse spectrophotometer. Fluorescently labeled FAM-NP-3C (1 µM, 100 µL) was diluted in PBS, pH 7.4 (100 µL), and the fluorescence emission was recorded between 495 - 750 nm using an excitation wavelength of λ_{ex} = 495 nm. Likewise, the fluorescence emission of Cy3-tagged $PMPC_{25}$-$PDPA_{70}$ polymersomes (2.5 mg mL^{-1}) and a mixture of Cy3-tagged $PMPC_{25}$-$PDPA_{70}$ polymersomes and DNA nanopores (2.5 mg mL^{-1} and 500 nM, respectively) was recorded between 495 nm to 750 nm with λ_{ex} = 495 nm.

1.11. Encapsulation of Trypsin into Polymersomes, their Purification by Size Exclusion Chromatography and Characterization by UV-vis Spectroscopy

Porcine trypsin (1000-2000 U/mg) was encapsulated into polymersomes by electroporation following a published procedure.[8] Briefly, a suspension of $PMPC_{25}$-$PDPA_{72}$ polymersomes (5 mg mL^{-1}, 200 µL) and a solution of trypsin (25 mg mL^{-1}, 200 µL) were gently mixed and placed into an electroporation cell. This mixture was subsequently transferred in the electroporator (Eppendorf 2510) and electroporated using 5 pulses of an AC electric field at a voltage of 2500 V with an interval of 30 s between each pulse.

Polymersomes with encapsulated trypsin were purified from excess free trypsin by SEC using Sepharose 4B. The gel filtration medium (20% slurry in EtOH) was first washed multiple times with PBS and centrifuged (5000 RCF, 5 min). Sepharose was then packed into the chromatographic column and washed 5 times with PBS. The sample (400 µL) was applied to the column, and purified material was eluted by collecting 500 µL fractions. Isolated trypsin and polymersomes that was not exposed to trypsin were also purified to obtain the corresponding reference elution volumes (10 - 15 mL and 5 - 6 mL, respectively).

The protein content in the SEC-purified polymersome fraction containing encapsulated trypsin was analyzed by UV-vis spectroscopy to obtain absorbance readings at 280 nm using a Carry Eclipse Varian spectrophotometer. To determine the $PMPC_{25}$-$PDPA_{72}$ polymer content, protein-free polymersomes that had been subjected to the same purification procedure as polymersomes with encapsulated trypsin were analyzed by UV-spectroscopy at 220 nm. The polymersome solution (20 µL) was diluted 10-fold in PBS at pH 2.0. The concentration of trypsin was $PMPC_{25}$-$PDPA_{72}$ was calculated from absorbance readings and calibration curves for $PMPC_{25}$-$PDPA_{72}$ polymer and trypsin in PBS at pH 2.0. The polymer concentration was subsequently used to calculate the number of polymersomes as described in section 1.5.

1.12. Enzymatic Assays of Polymersomes with Encapsulated Trypsin

Nanoreactor assays were carried out with SEC-purified polymersomes containing encapsulated trypsin. A suspension of polymersomes (14 µM trypsin, 0.62 mg mL^{-1} polymer, 50 µL) and solutions of DNA nanopores NP-3C or NP-0C (1 µM, 25 µL) and B-NAR-AMC peptide (1 mM, 25 µL) were added to PBS, pH 7.4 (100 µL). For the negative control, the DNA nanopore solution was replaced with PBS, pH 7.4 (25 µL). For the positive control without polymersomes and DNA nanopores, B-NAR-AMC peptide (1 mM, 25 µL) and trypsin solution (25 µM, 50 µL) were mixed with PBS, pH 7.4 (125 µL). To avoid immediate reaction in the positive control, a lower concentration of trypsin (500 nM, 50 µL) was used to lower the conversion rate. All measurements were recorded on a Carry Eclipse fluorescence spectrophotometer. The fluorescence emission of each mixture was monitored between 400 and 600 nm with λ_{exc} = 380 nm.

2. Experimental Results

2.1. Characterization of Polymersomes

Scheme S1: Schemes for the synthesis of (A) PMPC-PDPA and (B) PDPA-PMPC-S-S-PMPC-PDPA. (C) Scheme for labeling of PMPC-PDPA with Cy3.

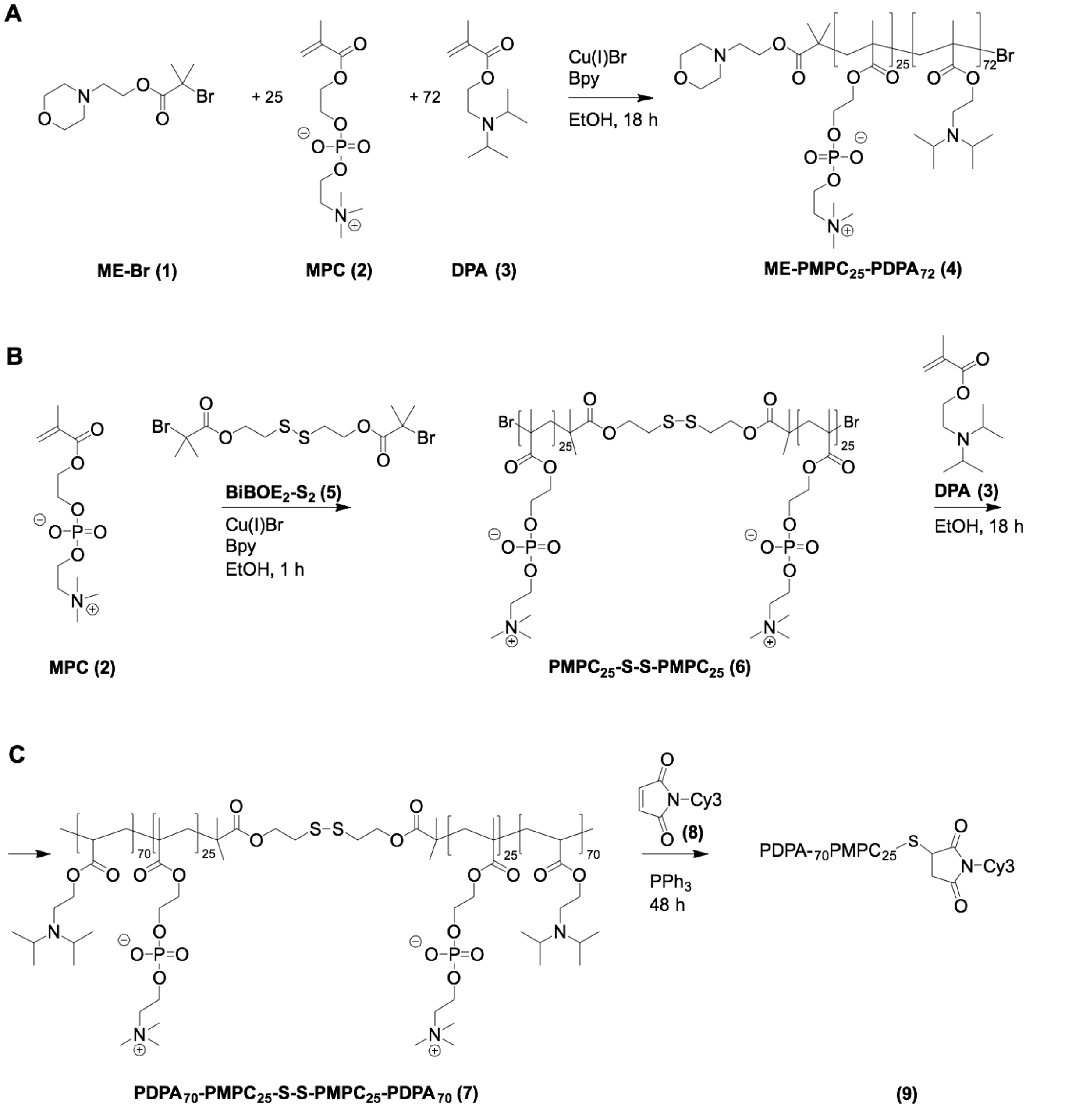

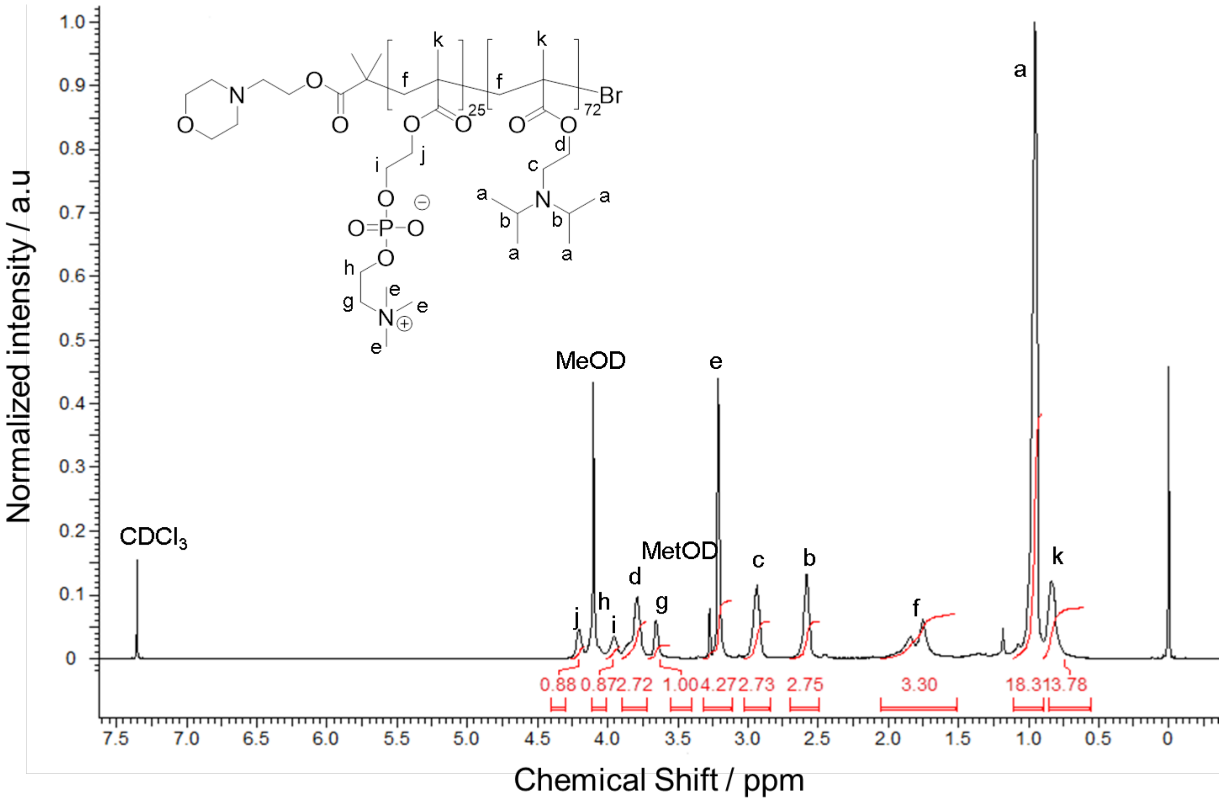

Figure S2. ^{1}H NMR spectrum of PMPC-PDPA (3) (600 MHz, $CDCl_3$:MeOD, 3:1) δ: 0.80 (broad peak **k**, 3H, −(CH_3)), 0.95 (doublet **a**, 12H, C**H₃**−CH−C**H₃**), 1.50-1.90 (broad peaks **f**, backbone), 2.55 (broad peak **b**, 2H, CH_3−C**H**−CH_3), 2.95 (broad peak **c**, 1H, −O−CH_2−C**H₂**−N−), 3.20 (singlet **e**, 9H, C**H₃**−N−), 3.65 (broad peak **g**, 2H, −P−O−CH_2−C**H₂**−N−), 3.80 (broad peak **d**, 2H, −O−CH_2−C**H₂**−N−), 3.90 (broad peak **i**, 2H, −O−CH_2−C**H₂**−O−P−), 4.05, 4.20 (broad peak **h**, 2H, −P−O−C**H₂**−CH_2−N−), 4.25 (broad peak **j**, 2H, −O−C**H₂**−CH_2−O−P−), composition $PMPC_{25}$-$PDPA_{72}$.

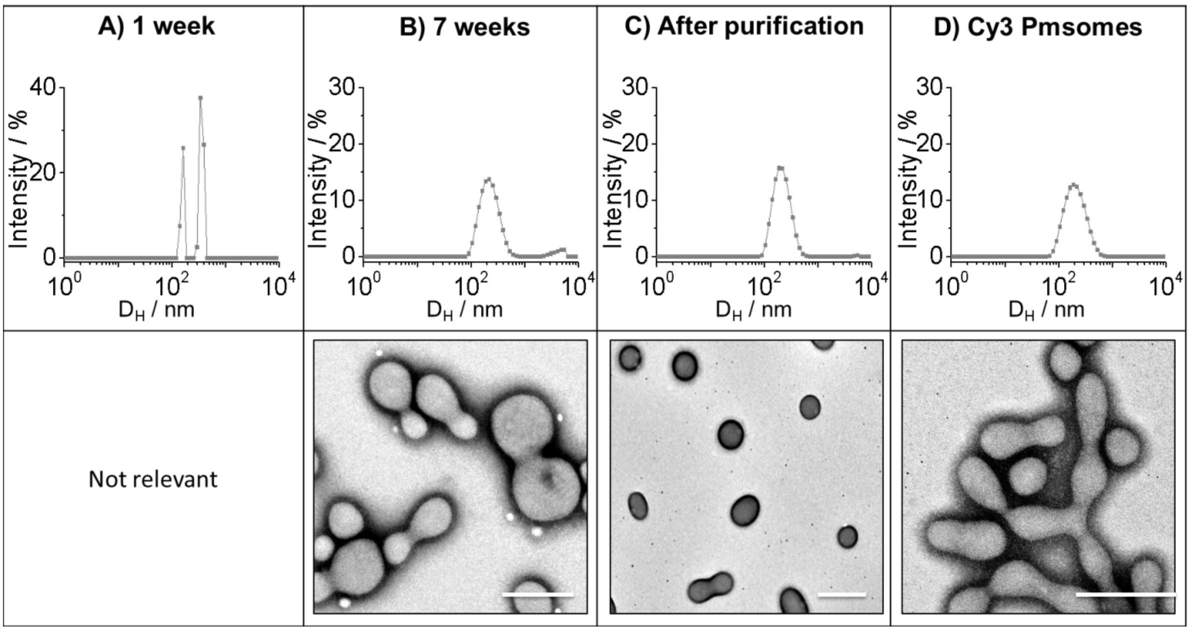

Figure S3. Analysis of polymersomes at different stages of their preparation as determined with DLS and TEM. Analysis (A) after one week of hydration under stirring, (B) after seven weeks of hydration under stirring, (C) after purification via repeated centrifugation and washing to pellet large, non-spherical, aggregating polymersomes, and (D) of the polymersome dispersion of a 5:95 mixture of Cy3-$PMPC_{25}$-$PDPA_{70}$ and $PMPC_{25}$-$PDPA_{72}$ block copolymers prior to purification. Unpurified polymersomes are of heterogeneous and non-spherical shape; purification by centrifugation leads to their aggregation leaving spherical vesicles in suspension. Samples with a polymer concentration of 0.5 mg mL^{-1} were adsorbed onto EM grids, followed by staining with PTA. Scale bars, 200 nm.

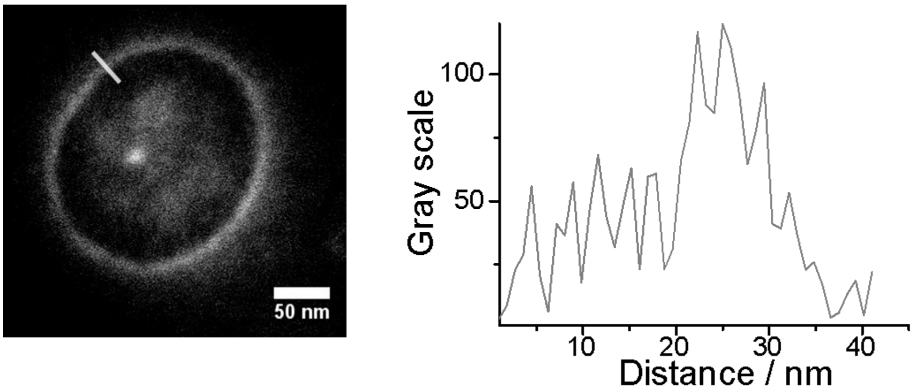

Figure S4. Scanning transmission electron microscopy image analysis of polymersomes to determine membrane thickness and a corresponding plot profile of thickness. An aliquot of a $PMPC_{25}$-$PDPA_{72}$ polymersome sample (polymer concentration 0.5 mg mL^{-1}, 5 μL) was deposited onto a freshly glow-discharged carbon-coated copper grid and subsequently stained with PTA. The brightness in the dark-field image represents electrons diffracted by tungsten of the PTA stain. The thickness was obtained from the FWMH of 10 plot profiles of brightness yielding a value of 6.5 ± 1.2 nm. This accounts for both PMPC and PDPA blocks.

2.2. Characterization of DNA Nanopores

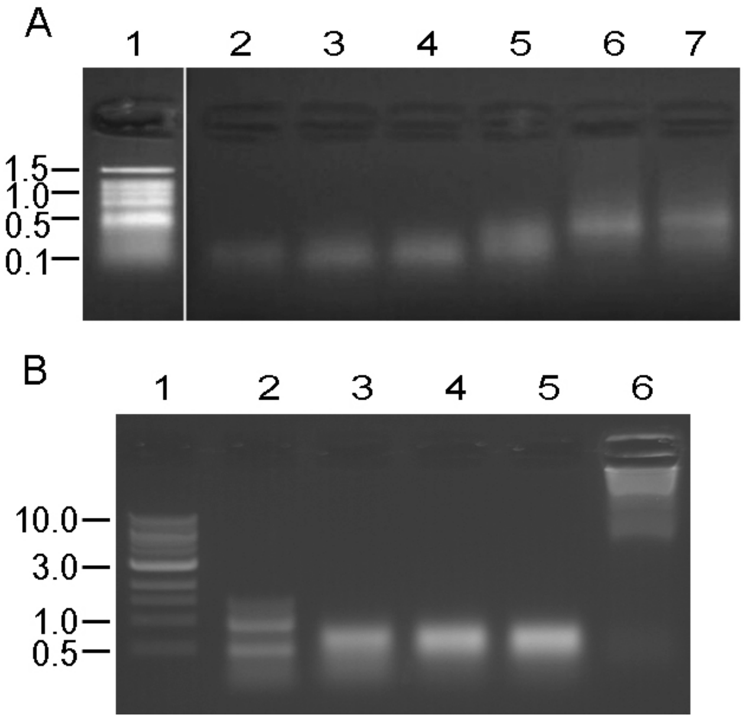

Figure S5. Agarose gel analysis of DNA nanopores and component DNA strands. (A) 2% agarose gel. Lane 1, 100 bp marker; lane 2, strand 1; lane 3, strands 1-2; lane 4, strands 1-3; lane 5, strands 1-4; lane 6, strands 1-5; lane 7, NP-0C. (B) 1.2 % agarose gel. Lane 1, 1 kbp marker; lane 2, 100 bp marker; lane 3, NP-0C 2 months old; lane 4, NP-0C 1 month old; lane 5, NP-0C fresh sample; lane 6, NP-3C fresh sample.

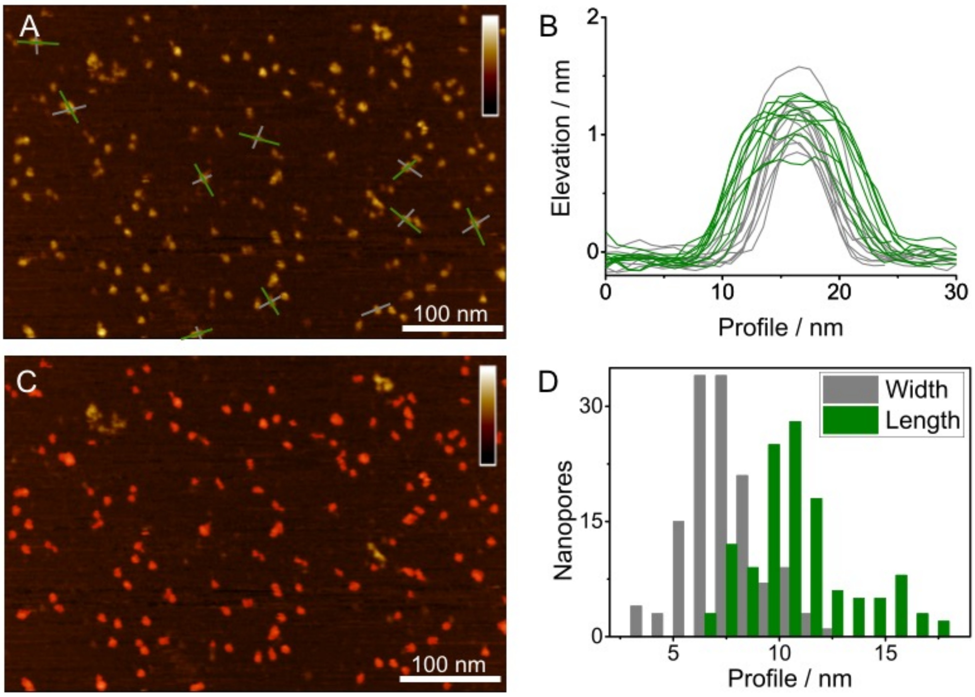

Figure S6. AFM analysis of DNA nanopore NP-0C adsorbed on mica using $NiCl_2$. (A,C) AFM micrographs. Vertical scale 2.5 nm (inset). Micrograph (A) highlights which pores were manually selected to obtain elevation profiles for nanopore length (green) and nanopore width (grey) as plotted in (B). Micrograph (C) displays the singular pores selected by a elevation threshold of the analysis software to determine the distributions of nanopore width and length as plotted in (D). The dimensions determined for nanopore length and width by each method are: (B) 11.4 ± 4.3 nm and 6.0 ± 2.5 nm respectively, (FWHM, n = 10), and (D) 11.3 ± 3.2 nm and 7.6 ± 1.7 nm respectively (n = 131).

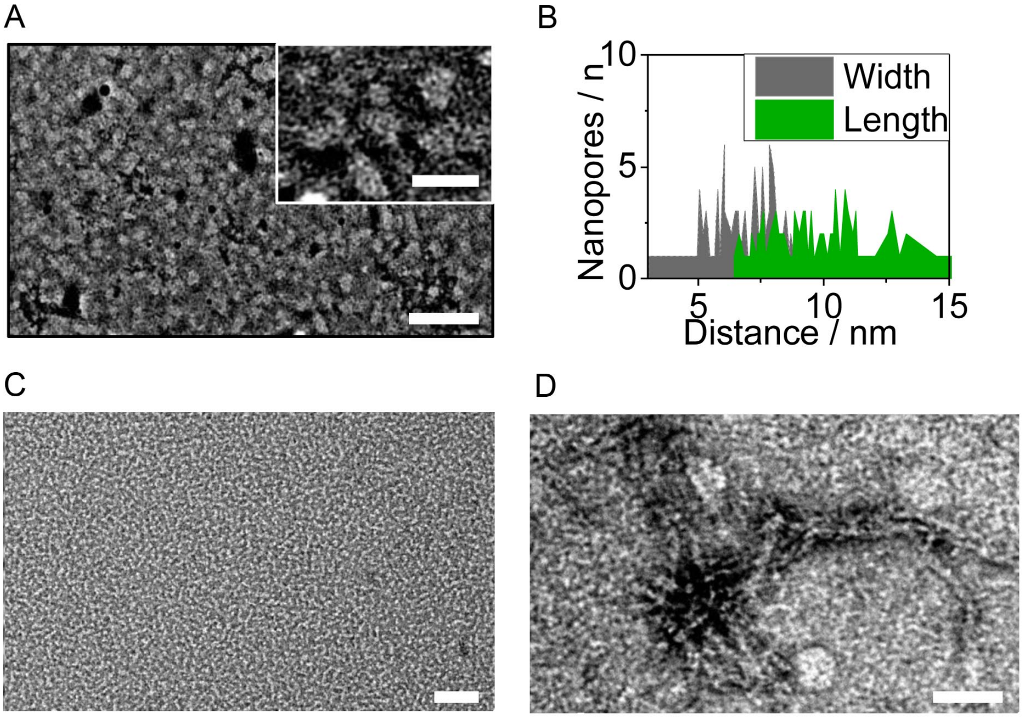

Figure S7. Characterization of NP-3C DNA nanopores via TEM. (A) TEM image of UA-stained NP-3C adsorbed on EM grids (droplet of 5 nM, 5 µL)(scale bar, 50 nm; inset, 20 nm). The elongated bright spots are interpreted as DNA pores. (B) Analysis of spots reveals an average length and width of 9.9 ± 2.0 nm and 7.0 ± 1.4 nm (n = 100) respectively, in agreement with pore's nominal dimensions (9 nm height and 5.5 nm width). (C) TEM image of EM grids after UA stain but without DNA pores (scale bar, 50 nm). The graininess in the image is considerably smaller than in panel A which supports the interpretation of bright spots in A as DNA pores. (D) TEM image for UA-stained reference DNA nanopores of the same 6-helix bundle architecture as NP-3C but a length of 15 nm[9]. The tubes confirm that the UA staining procedure can successfully visualize DNA pores. Elongated tubes rather than shorter 15 nm long structures appear because the reference pore, unlike NP-3C, feature a shortened duplex hairpin loops that enable base π-stacking between head-to-tail assembled pores[5,9] (scale bar, 50 nm).

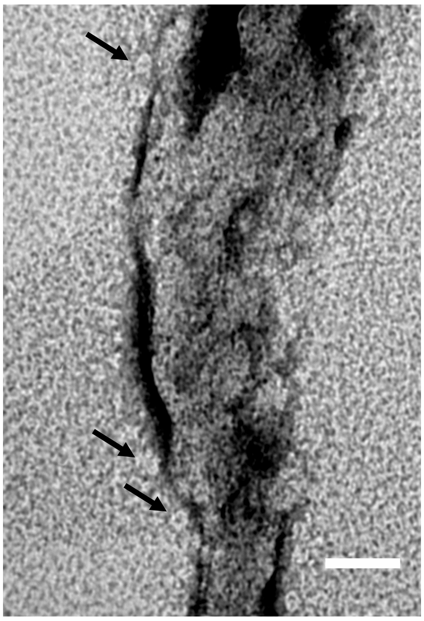

Figure S8. TEM micrograph of NP-3C (adsorption from a droplet, 1 nM, 5 μL) stained with UA. The image displays fewer pores than in the analysis of pores from a solution of 5 nM shown in Figure S7. Scale bar, 50 nm.

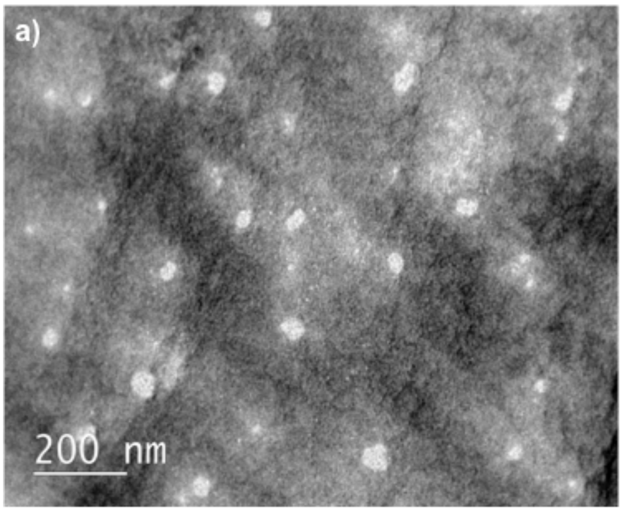

Figure S9. TEM micrographs of DNA nanopores NP-3C (10 nM, 10 µL) stained with AM. Scale bar, 200 nm.

2.3. Characterization of Hybrid Nanocontainers Composed of Polymersomes and DNA Nanopores

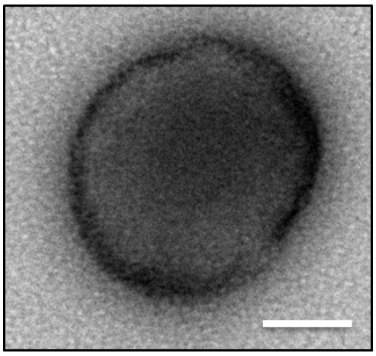

Figure S10. TEM micrograph of a polymersome incubated with a solution of anchor-free DNA nanopore NP-0C. The mixture contained polymersomes (0.5 mg mL^{-1}, 5 µL), NP-0C (10 nM, 10 µL) and 0.2 x PBS, 7.4 (35 µL) and was incubated for 15 min, and 5 µL were deposited on the grid and stained with AM. Scale bar, 50 nm.

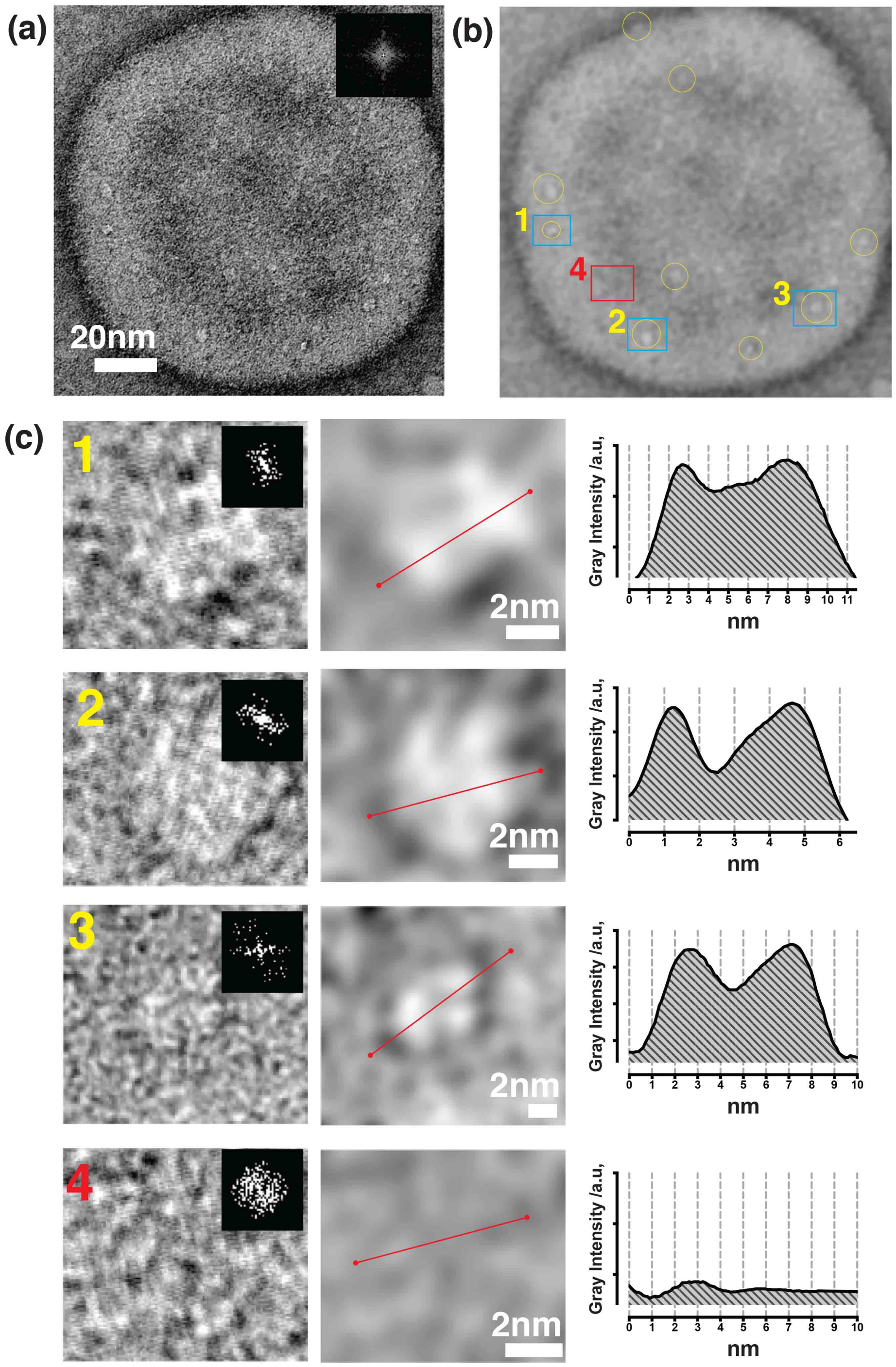

Figure S11. TEM analysis of DNA pores anchored into polymersome membranes. The concentrations of DNA pores and polymersomes in the incubation mixture deposited onto the EM grid were 50 nM and 0.5 mg mL^{-1}, respectively. (A) Raw image of a polymersome with inserted NP-3C nanopores. The inset on the top right is the image after fast Fourier transform (FFT) filtering. (B) FFT filtered image to highlight and identify inserted nanopores. (C) Different regions of interest (ROI) shown as raw image (left), FFT-image (middle), and in cross-sectional profile (right) along the red line in the FFT image. The ROIs 1-3 show pores while ROI 4 displays the polymersome membrane without pore.

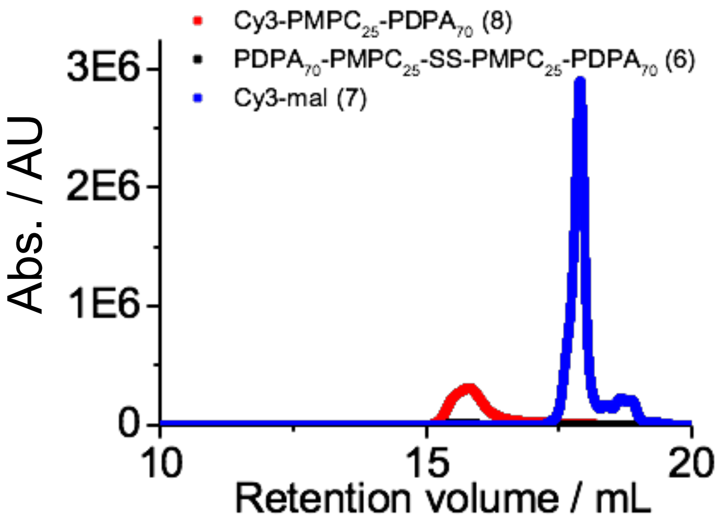

Figure S12. HPLC traces of Cy3-labeled $PMPC_{25}$-$PDPA_{70}$ (**9**, Scheme 1), $PDPA_{70}$-$PMPC_{25}$-SS-$PMPC_{25}$ $PDPA_{70}$ (**7**, Scheme 1), and Cy3-mal (**8**, Scheme 1) recorded at an absorption wavelength of 568 nm. HPLC conditions are given in chapter 1.4.

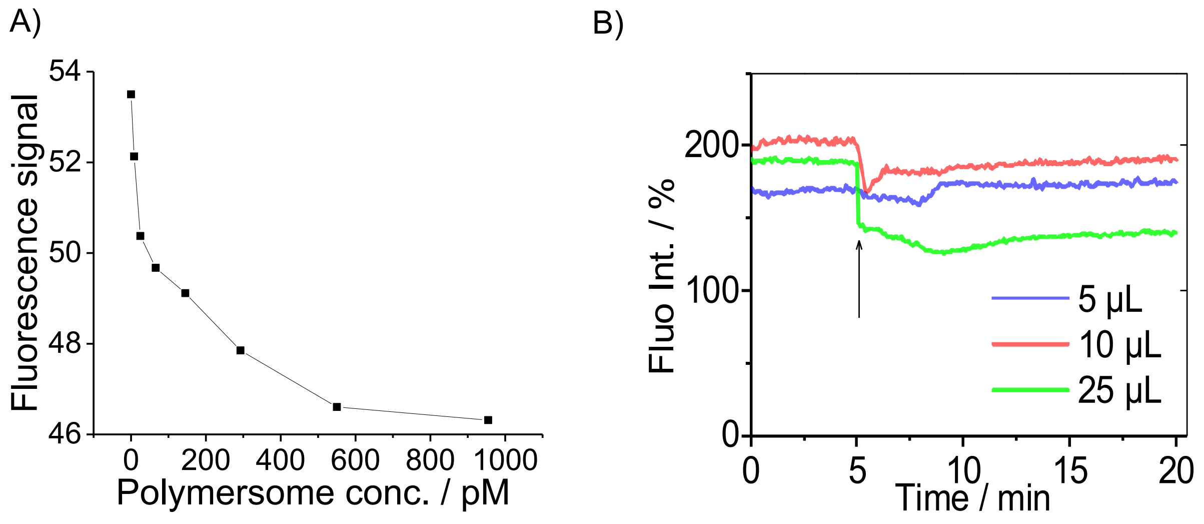

Figure S13. Varying the molar ratio of DNA nanopore and polymersome in the incubation mixture tunes the amount of membrane-inserted nanopores. A) Titration curve plotting the extent of fluorescence quenching of FAM-labeled NP-3C as a function of polymersome concentration. The concentration of NP-3C was constant at 50 nM in PBS, pH 7.4. Quenching is caused by inserting the FAM fluorophore into the polymersome wall. B) Fluorescence traces of FAM-labeled NP-3C (500 nM, 200 µL) after mixing (see arrow) with Cy3-labeled polymersomes suspensions (5 mg mL^{-1}) ranging in volume from 5 µL to 25 µL. A drop in fluorescence signal at λ_{em} = 520 nm corresponds to fluorescence quenching caused by anchoring of the NP-3C into the polymersome membrane.

2.4. Characterization of Nanocontainers with Encapsulated Enzymes

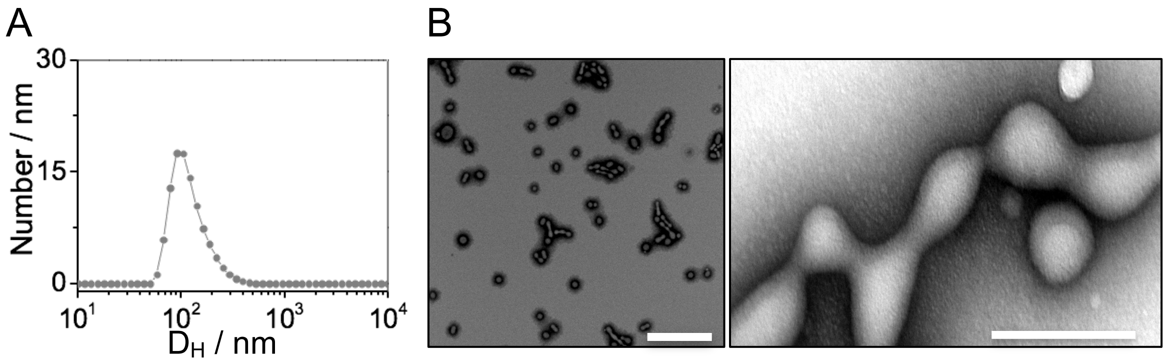

Figure S14. (A) DLS and (B) TEM analysis of a SEC-purified fraction of polymersomes containing encapsulated trypsin. TEM samples were stained with PTA. Scale bar for left panel, 1 µm; for right panel, 200 nm.

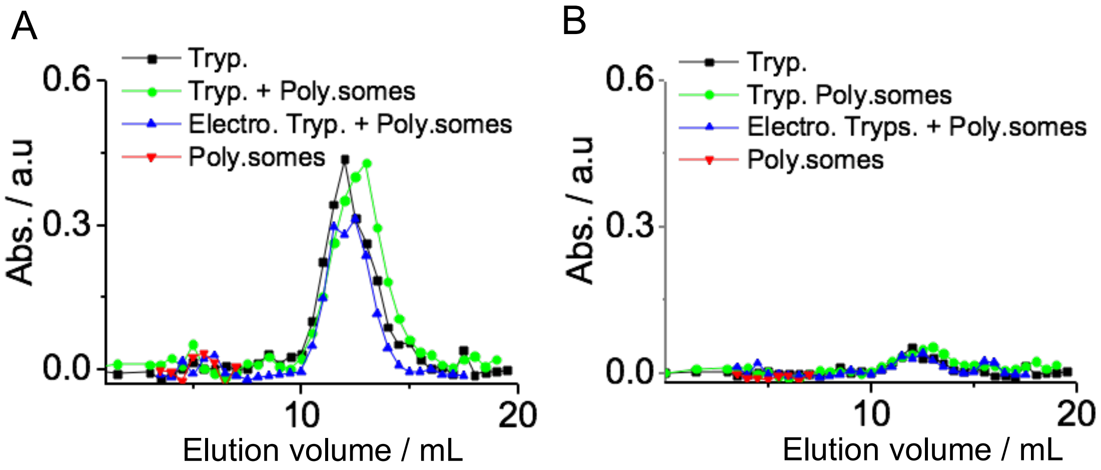

Figure S15. SEC-elution profiles of free trypsin (black), free trypsin and polymersomes (green), electroporated trypsin and polymersomes (blue), and polymersomes (red) using a Sepharose 4B column with a length of 18 cm. The absorbance was recorded at a wavelength of (A) 220 nm and (B) 280 nm.

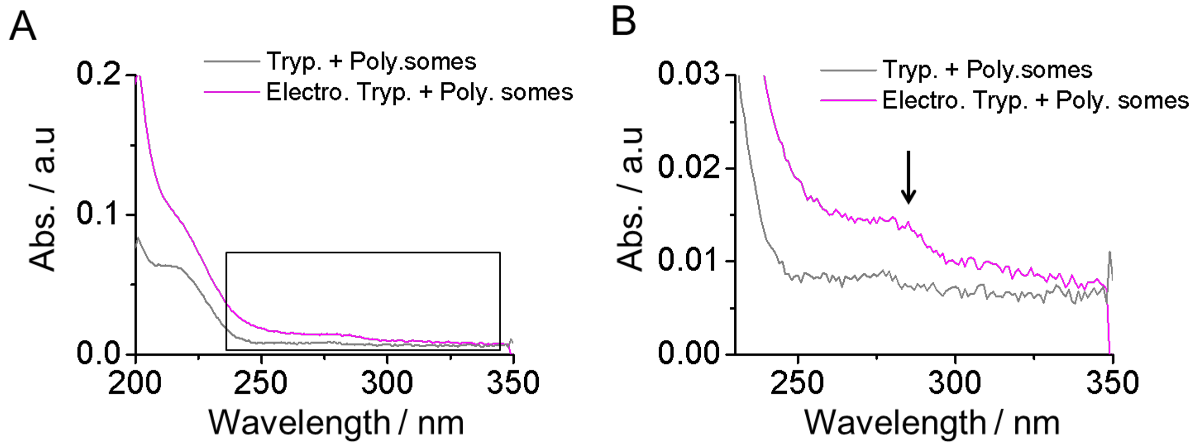

Figure S16. Absorbance spectra of denatured and SEC-purified polymersomes with trypsin subjected (purple) and not subjected to electroporation (grey). The sample was diluted 10 times in PBS, pH 2.0 before measurement. Panel (B) shows a zoom-in of the box in panel (A).

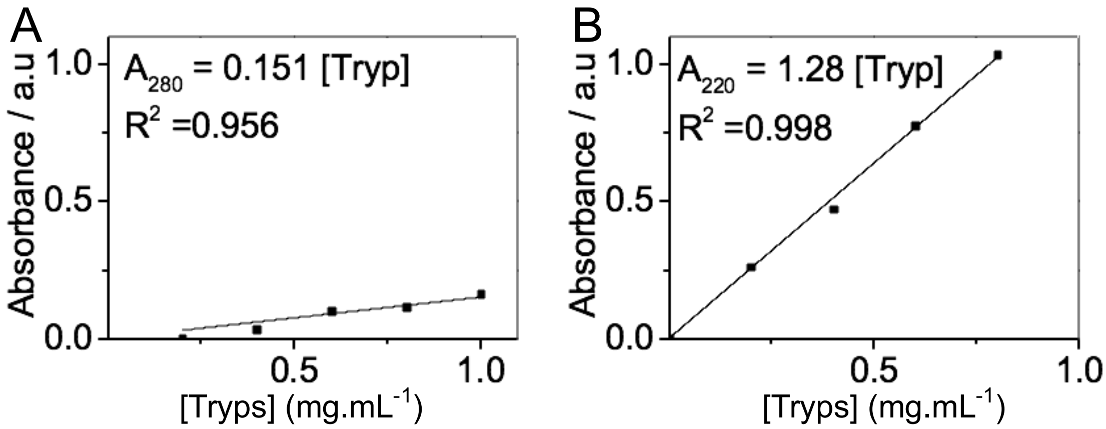

Figure S17. Concentration-dependent absorption of trypsin from porcine pancreas in PBS, pH 2.0. Absorbance was recorded at (A) 280 nm and (B) 220 nm.

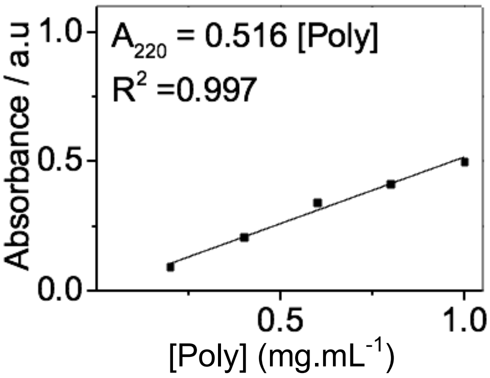

Figure S18. Calibration curve of $PMPC_{25}$-$PDPA_{72}$ block copolymer in PBS, pH 2.0. Absorbance was recorded at 220 nm.

2.5. Characterization of Hybrid Nanocontainers with Enzymatic Assays

Table S2: The molecular composition of nanoreactors quantified by the concentrations and ratios for polymersomes, DNA nanopores, enzyme, and peptide molecules in the enzymatic assay on hybrid nanoreactors.[1]

	**Polymer[2]**	**Polymersomes[3]**	**Nanopores[4]**	**Enzyme[5]**	**Peptide**
Concentration (μM)	7.04	0.16×10^{-3}	0.125	0.44	125
Moles	3.52×10^{-9}	7.8×10^{-13}	6.25×10^{-11}	2.20×10^{-10}	6.25×10^{-8}
Number	2.12×10^{15}	4.70×10^{11}	3.73×10^{13}	1.33×10^{14}	3.77×10^{16}
Ratio for one vesicle	4520	1	80	282	80100

[1]All values correspond to the concentration in nanoreactor assay mixture, as described in section 1.12. [2]The polymer concentration in the polymersome sample that was not subjected to electroporation was 0.16 mg mL^{-1}. The concentration was obtained from the absorbance reading of 0.06 at 220 nm (Figure S16) and a calibration curve (Figure S18). [3]The number of polymersomes was calculated from the polymersome size (DLS) and the polymer concentration, as described in section 1.5. [4]The number of DNA nanopores per vesicles only refers to ratio in the incubation mixture but not to the value of membrane-inserted pores. [5]The concentration of trypsin encapsulated within polymersome was calculated from the absorbance reading at 280 nm minus the baseline intensity calculated at 300 nm (Figure S16; calibration curve, Figure S17) obtained from the difference between the samples that were and were not electroporated (Figure S17).

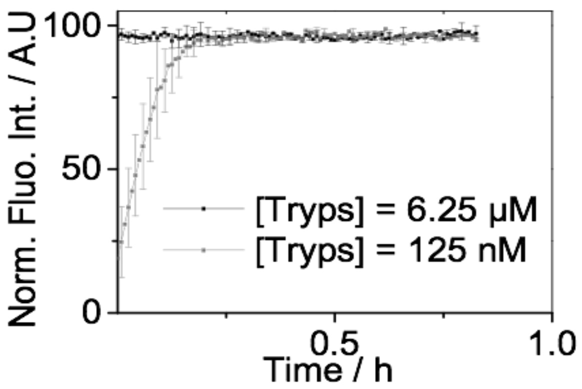

Figure S19. Kinetic fluorescence trace for the enzymatic reaction between free, non-encapsulated trypsin and substrate B-NAR-AMC.

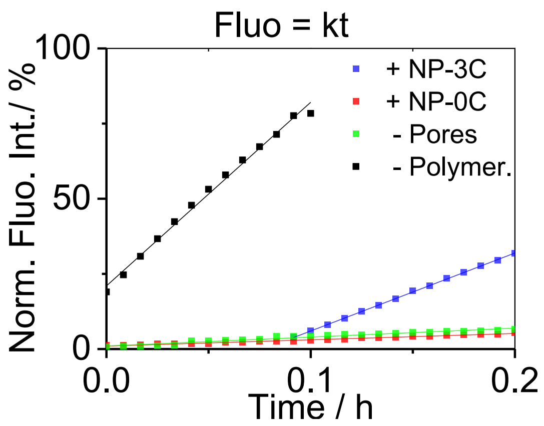

Figure S20: Linear fit to kinetic fluorescence traces of the nanocontainer with DNA nanopores NP-3C (blue), negative controls NP-0C (red) and without nanopores (green), and positive control without polymersomes (black). The molecular ratio of peptide : enzyme : polymersome : DNA-pore was 80 000 : 280 : 1 : > 7. The number of DNA pores is based on the TEM analysis in Figure 2 but is most likely higher as a 8-fold higher molar ratio of DNA pores to polymersomes was used in the incubation mixture for the enzymatic assay as compared to the preparation of samples for the TEM analysis.

Table S3: Fits to the enzymatic activity of nanoreactors as determined via fluorescence measurements of B-NAR-AMC hydrolysis.

Sample	Initial Rate in Fig. S21 (k / s^{-1})
+ NP-3C	259
+ NP-0C	21
no pores	30
Polymer (Free trypsin and B-NAR-AMC, at 6.25 μM)	610 x 50 = 30500

References:

[1] L. Ruiz-Perez, J. Madsen, E. Themistou, J. Gaitzsch, L. Messager, S. P. Armes, G. Battaglia, *Polym. Chem.* **2015**, *6*, 2065-2068.

[2] H. Lomas, I. Canton, S. MacNeil, J. Du, S. P. Armes, A. J. Ryan, A. L. Lewis, G. Battaglia, *Adv. Mater.* **2007**, *19*, 4238-4243.

[3] J. Gaitzsch, M. Delahaye, A. Poma, F. Du Prez, G. Battaglia, *Polym. Chem.* **2016**, *7*, 3046-3055.

[4] J. Gaitzsch, D. Appelhans, L. Wang, G. Battaglia, B. Voit, *Angew. Chem. Int. Ed.* **2012**, *51*, 4448-4451.

[5] J. R. Burns, A. Seifert, N. Fertig, S. Howorka, *Nat. Nanotechnol.* **2016**, *11*, 152-156.

[6] M. Bussiek, N. Mucke, J. Langowski, *Nucleic Acids Res.* **2003**, *31*, e137.

[7] D. Necas, P. Klapetek, *J. Physics* **2011**, *10*, 181–188.

[8] L. Wang, L. Chierico, D. Little, N. Patikarnmonthon, Z. Yang, M. Azzouz, J. Madsen, S. P. Armes, G. Battaglia, *Angew. Chem. Int. Ed.* **2012**, *51*, 11122-11125.

[9] J. Burns, E. Stulz, S. Howorka, *Nano Lett.* **2013**, *13*, 2351-2356.
